# Strengthening Biosecurity in Iraq: Development of a National Biorisk Management System

**DOI:** 10.3389/fpubh.2016.00025

**Published:** 2016-02-26

**Authors:** Mahdi F. H. Al Jewari, Gregory D. Koblentz

**Affiliations:** ^1^Iraqi National Monitoring Authority, Ministry of Science and Technology, Baghdad, Iraq; ^2^School of Policy, Government, and International Affairs, George Mason University, Fairfax, VA, USA

**Keywords:** Iraq, biosecurity, public health, bioengagement, biodefense, global health, biosafety, biorisk

## Abstract

Since 2004, the Republic of Iraq has undertaken a concerted effort to comply with all of its international obligations to prevent the proliferation and the use of chemical, biological, radiological, and nuclear (CBRN) weapons. A centerpiece of this effort is Iraq’s development of a National Biorisk Management System. The Iraqi National Monitoring Authority (INMA), which is responsible for CBRN security and non-proliferation in Iraq, has played a key role in establishing this system. This article provides an overview of Iraq’s international non-proliferation commitments, describes the legal and organizational steps it has taken to implement these commitments, and examines current initiatives to strengthen Iraq’s biosecurity.

## Iraq’s Implementation of Its Non-Proliferation Commitments

Since 2004, Iraq has strengthened its commitment to the non-proliferation of CBRN weapons. Iraq is now party to all of the major international non-proliferation treaties (see Table 1). Iraq welcomed the adoption of United Nations Security Council Resolution (UNSCR) 1540 and submitted its first National Report to the 1540 Committee on April 13, 2005. In 2014, Iraq submitted a report to the committee on its experiences, best practices, and lessons learned from implementation of UNSCR 1540 (1).

**Table 1:** **Iraq’s non-proliferation commitments**.

Treaty	Date of signature	Date of ratification or accession
Geneva Protocol		September 8, 1931
Partial Test Ban Treaty	August 13, 1963	December 3, 1964
Nuclear Non-Proliferation Treaty (NPT)	July 1, 1968	October 29, 1969
Biological Weapons Convention (BWC)	May 11, 1972	June 19, 1991
Comprehensive Nuclear-Test-Ban Treaty (CTBT)	August 19, 2008	September 26, 2013
IAEA’s Additional Protocol	October 9, 2008	October 10, 2012
Chemical Weapons Convention (CWC)		January 13, 2009
Hague Code of Conduct (HCOC)	June, 2011	June, 2011
Convention on the Physical Protection of Nuclear Material		July 7, 2014

Iraq has taken a series of practical steps to implement its obligations under international non-proliferation treaties to prevent the proliferation of weapons of mass destruction and their means of delivery to states and non-state actors. Article 9, paragraph e of Iraq’s constitution, adopted by popular referendum in 2005, states that: “the Iraqi government shall respect and implement Iraq’s international obligations regarding the non-proliferation, non-development, non-production and non-use of nuclear, chemical and biological weapons and shall prohibit associated equipment, material, technologies and delivery systems for use in the development, manufacture, production, and use of such weapons.”

In 2006, Iraq began developing national legislation to implement and enforce its treaty and constitutional commitments to prevent the proliferation of CBRN weapons and their means of delivery. The draft law was reviewed by the International Atomic Energy Agency (IAEA) and the Organization for the Prohibition of Chemical Weapons (OPCW), the international organization in charge of implementing the Chemical Weapons Convention (CWC). The Iraqi parliament passed the National Monitoring Authority for Non-Proliferation Act No. 48 (2012) on February 16, 2012 and the legislation went into effect on September 1, 2012. The Act prohibits the development, production, possession, transfer, or use of CBRN weapons and their means of delivery and establishes a system to control the import, export, and transfer of dual-use materials. Violations of the law can be punished by fines of up to 200 million Iraqi dinars and life imprisonment.

## Iraqi National Monitoring Authority

The National Monitoring Authority for Non-Proliferation Act No. 48 (2012) established the INMA, part of the Ministry of Science and Technology, as the main agency charged with implementing the new legislation. INMA was formed on the basis of the National Monitoring Directorate that had been created in 1993 to serve as Iraq’s official liaison with the IAEA and the United Nations Special Commission (UNSCOM), the organization charged by the United Nations Security Council after the 1991 Persian Gulf War with overseeing Iraq’s disarmament in the fields of missiles and chemical and biological weapons. INMA’s overarching goal is to establish and maintain a national system of monitoring, investigation, and inspection that enables Iraq to comply with its national and international non-proliferation obligations. INMA is organized into five main functional departments (Nuclear, Biological, Chemical, Means of Delivery, and Import/Export) and a number of supporting units (Operations, Research and Studies, Administrative, Legal and Financial, Public and International Relations, and Chairman’s Office).

Iraqi National Monitoring Authority has three primary functions: compliance, monitoring of dual-use materials, and capacity building. First, INMA is the lead agency for interacting with international non-proliferation organizations and is responsible for ensuring Iraqi compliance with its international non-­proliferation commitments. To achieve this objective, INMA is the designated point of contact for the OPCW, the BWC’s Implementation Support Unit (ISU), the IAEA, the 1540 Committee, and the Comprehensive Test Ban Treaty Organization. INMA is also responsible for preparing declarations and reports required by these organizations and accompanies inspection teams from these organizations during their visits to Iraq. In the context of the BWC, INMA compiles and submits annual confidence-building measure reports to the ISU and participates in meetings of experts, meetings of state parties, and review conferences (2).

Iraqi National Monitoring Authority’s second major function is to develop regulations and mechanisms to monitor the production, use, storage, import, export, and shipping of dual-use materials to ensure their safe, secure, and peaceful application in accordance with national and international laws and standards. INMA, in cooperation with other Iraqi ministries, has developed a national system to control the import, export, and movement of dual-use materials. Iraq’s list of controlled dual-use materials is based on lists maintained by the Missile Technology Control Regime, Nuclear Suppliers Group, Australian Group, Wassenaar Arrangement, and EU Law No. 2000/1334. All Government institutions and private sector companies and individuals who intend to import or export dual-use items must obtain a license from the Ministry of Trade (MOT). MOT is responsible for checking the accuracy of the information and forwarding the information to INMA for registration. If INMA grants permission for the requested license, MOT sends a copy of the license to the General Authority for Customs (GAC). GAC matches the shipping manifests of dual-use materials being transferred with the information in the license and informs its inspectors at border crossings who are responsible for checking the imported or exported materials against the license. If the license is for an import, INMA conducts a follow-up inspection with the end user of the material and makes any declaration required under international non-proliferation treaties. The Ministry of Transportation issues instructions regarding the transfer of chemical, biological, radioactive, and hazardous materials, ensures carriers comply with safety and security standards, and reports any transfers or retransfers of dual-use materials to INMA.

Iraqi National Monitoring Authority’s third major task is to develop and implement capacity building programs to enhance Iraq’s ability to prevent the proliferation of CBRN weapons to states and non-state actors and strengthen Iraq’s preparedness for responding to the use of such weapons. To fulfill this function, INMA has started to hold workshops for managers and staff of different Iraqi ministries to raise awareness of the threat of CBRN weapons, the identification of dual-use materials, and implementation of UNSCR 1540. INMA has also launched several projects, in cooperation with foreign partners, including the United States, Switzerland, Netherlands, Norway, Germany, and Jordan, to enhance Iraq’s capacity to detect and respond to man-made and naturally occurring biological threats.

## Initiatives to Strengthen Biosecurity in Iraq

In 2012, INMA proposed the creation of a high-level interagency coordinating body to create a comprehensive National Biorisk Management System in Iraq. INMA worked with the Public Health Directorate of the Ministry of Health to identify institutions that should be represented on this committee and to determine the committee’s goals for strengthening Iraq’s preparedness for natural and man-made disease outbreaks. On May 1, 2012, the General Secretariat of the Council of Ministers issued order No.18778 which established the National Biorisk Management Committee (NBMC). The NBMC is chaired by the Ministry of Health and is composed of representatives from the following ministries: Agriculture, Defense, Interior, Higher Education, Finance, Planning, Industry, Prime Minister’s Council, Science and Technology, Environment, Prime Minister’s Advisory Commission, Trade, and Water Resource and Irrigation. Additional ministries participate as is necessary.

The NBMC has identified four priorities for strengthening Iraq’s national capacity to counter biological threats: establishing a national pathogen list, building laboratory capacity, developing the capability to conduct joint law enforcement–public health investigations, and establishing a biorisk management law. The NBMC has established sub-committees charged with developing new policies and programs to achieve these four objectives.

### National Pathogen List

The NBMC is leading the development of a comprehensive list of human, plant, animal, and zoonotic pathogens that will serve multiple purposes in Iraq’s National Biorisk Management System. The national pathogen list will become part of the system regulating the import, export, and transfer of dual-use materials. In addition, the list will be used to determine appropriate biosafety and biosecurity measures that laboratories will need to implement. Finally, the list will be used to guide bioterrorism preparedness and response activities. A subcommittee of the NBMC, which is composed of INMA, the Ministry of Public Health, and the Ministry of Agriculture, has worked on developing the list since the beginning of 2014. The subcommittee has used pathogen lists developed by other countries and international organizations, including the United States, European Union, and Australia Group, as a starting point for its work. However, these lists are shaped by national security, public health, agricultural, and economic factors unique to each country and organization. For example, foot-and-mouth disease (FMD) is viewed as a top bioterrorist threat by the United States due to the high vulnerability of its cattle to this disease and the severe economic consequences that an FMD outbreak would have (3). By contrast, FMD is endemic in Iraq and to cope with recurrent major FMD outbreaks, Iraq has developed a robust veterinary and vaccine response strategy (4). Therefore, imposing tight biosecurity limits on FMD virus would not make sense for Iraq. Instead of incorporating an existing pathogen list directly into its National Biorisk Management System, the subcommittee has sought to identify key criteria used to develop these lists and adapt them to Iraq’s unique situation. During the spring of 2015, one of the authors served as an CRDF Global Iraqi Bioscience Fellow at George Mason University where he evaluated the criteria used by the United States and European countries to assess and rank the safety and security risks posed by different pathogens. Based on this evaluation and other biorisk assessment exercises, the NBMC is considering applying the following criteria to generate its national pathogen list: pathogenicity, mode of transmission, route of exposure, level of morbidity and mortality, degree of antibiotic resistance, local availability of effective treatments and preventive measures, local diagnostic capabilities, potential economic losses caused by animal and plant agents, and the potential impact on the health-care system.

### Building Laboratory Capacity

A strong public health laboratory system is the foundation for an effective defense against naturally occurring infectious diseases and bioterrorism. The Ministries of Health and Agriculture maintain networks of laboratories to conduct surveillance, detection, and diagnosis of human, animal, and plant disease outbreaks (5). These ministries operate laboratories at biosafety level 1 and 2, which limits Iraq’s capability to safely conduct diagnostic tests on viruses such as highly pathogenic H5N1 avian influenza (6). Since 2009, the INMA has engaged in cooperative programs with Switzerland and the United States to rebuild Iraq’s public health laboratory capacity with the ultimate goal of establishing a biosafety level 3 laboratory. Iraqi officials and scientists from the Ministries of Agriculture, Health, and Science and Technology have received training on biosafety, biosecurity, emergency preparedness and response, and biorisk mitigation. Iraq has received assistance from the United States in the areas of disease surveillance, detection, diagnosis, biosafety, and biosecurity. To guide this work, INMA established an interagency technical working group to review Iraq’s biosurveillance capabilities, identify gaps, and propose solutions. The United States is in the process of upgrading the equipment at Iraqi public health and veterinary labs and linking provincial-level labs into a nation-wide network to improve Iraqi biosurveillance capabilities for human and animal disease outbreaks. This initiative helps Iraq meet its commitment under the 2005 International Health Regulations to develop core capacities in laboratory services and disease surveillance. In addition, the United States has provided some Iraqi laboratories with upgrades to their physical security, including the installation of electronic door locks to prevent unauthorized access to pathogen collections. Areas in need of further investment include improving biological waste management systems at public health labs, developing education and training curriculum, standardizing laboratory protocols, and developing an indigenous capability to maintain and repair biosafety equipment.

### Public Health–Law Enforcement Investigation

The potential for terrorists to employ CBRN weapons poses new challenges to both law enforcement and public health. Of all of the CBRN threats, biological weapons are the most difficult to investigate because of the potentially long delay between when an attack occurs and when the government becomes aware that an attack has taken place. Since it can be difficult to initially determine if a suspicious outbreak is natural or deliberate, public health authorities need to know when and how to contact law enforcement agencies if they suspect an outbreak is not natural. Likewise, law enforcement agencies need to be aware of the types of information that could indicate a terrorist group is interested in, has acquired, or has released biological agents and have procedures for sharing such information with public health authorities. Once it has been confirmed that an attack has occurred, public health and law enforcement agencies will need to coordinate their investigations to ensure that they collect and share the right information in a timely manner (7). The Ministry of Interior (MoI), which is in charge of investigating acts of terrorism, does not have a history of working with either the Ministry of Health or Ministry of Agriculture. Although MoI has extensive experience dealing with conventional improvised explosive devices and explosive ordnance disposal, it does not have any capacity for dealing with CBRN weapons or contaminated evidence. INMA is working to establish a joint public health–criminal investigative capacity in Iraq by holding interagency meetings, workshops, and training sessions to develop plans and procedures for information sharing and investigation in the event of a CBRN incident.

### Biorisk Management Law

Iraq lacks a legal framework for regulating the full range of biological risks, including disease outbreaks caused by natural sources, terrorists, and laboratory accidents, which threaten Iraq’s health and security. The NBMC has formed a subcommittee headed by INMA to draft a new law to provide a legal umbrella for the implementation of biorisk management policy in Iraq, including the national pathogen list, associated biosafety and laboratory security regulations, and the roles and responsibilities of different Iraqi ministries in strengthening Iraq’s preparedness for natural and deliberate disease threats. The NBMC’s goal is to issue the new law within 2–3 years.

## Conclusion

Since 2004, Iraq has made significant progress in improving its capacity to prevent the proliferation of CBRN weapons and their means of delivery in accordance with its legal, constitutional, and treaty obligations. Iraqi efforts to strengthen its preparedness for natural and deliberate disease threats has received valuable assistance from foreign partners who have engaged in collaborative capacity building activities over an extended period of time. A major challenge facing Iraq is the need to sustain these capabilities over time, especially if foreign assistance declines. Iraq will need to continue making investments in infrastructure, information technology, and human capital to ensure that its biosafety, biosecurity, and biosurveillance systems remain effective. The INMA has played a key role in Iraq’s development of a National Biorisk Management System, but achieving the four objectives established by the NMBC will require further interagency cooperation among Iraqi ministries as well as continued support from Iraq’s foreign partners. Only by working together can Iraq and the international community reduce the risks posed by infectious diseases, bioterrorism, and the misuse of biology.

## Author Contributions

All authors listed, have made substantial, direct and intellectual contribution to the work, and approved it for publication.

## Conflict of Interest Statement

MA was a Visiting Fellow at George Mason University during the Spring 2015 semester on a CRDF Global Iraqi Bioscience Fellowship. GK has no conflict of interest to declare.
